# Acceptance of voice assistant technology in dental practice: A cross sectional study with dentists and validation using structural equation modeling

**DOI:** 10.1371/journal.pdig.0000510

**Published:** 2024-05-14

**Authors:** Spencer Warren, Daniel Claman, Beau Meyer, Jin Peng, Emre Sezgin

**Affiliations:** 1 Department of Pediatric Dentistry, Nationwide Children’s Hospital, Columbus, Ohio, United States of America; 2 Division of Pediatric Dentistry, The Ohio State University College of Dentistry, Columbus, Ohio, United States of America; 3 Information Technology Research & Innovation, Nationwide Children’s Hospital, Columbus, Ohio, United States of America; 4 Center for Biobehavioral Health, The Abigail Wexner Research Institute, Nationwide Children’s Hospital, Columbus, Ohio, United States of America; 5 Department of Pediatrics, The Ohio State University College of Medicine, Columbus, Ohio, United States of America; University of Pittsburgh, UNITED STATES

## Abstract

Voice assistant technologies (VAT) has been part of our daily lives, as a virtual assistant to complete requested tasks. The integration of VAT in dental offices has the potential to augment productivity and hygiene practices. Prior to the adoption of such innovations in dental settings, it is crucial to evaluate their applicability. This study aims to assess dentists’ perceptions and the factors influencing their intention to use VAT in a clinical setting. A survey and research model were designed based on an extended Unified Theory of Acceptance and Use of Technology (UTAUT). The survey was sent to 7,544 Ohio-licensed dentists through email. The data was analyzed and reported using descriptive statistics, model reliability testing, and partial least squares regression (PLSR) to explain dentists’ behavioral intention (BI) to use VAT. In total, 257 participants completed the survey. The model accounted for 74.2% of the variance in BI to use VAT. Performance expectancy and perceived enjoyment had significant positive influence on BI to use VAT. Perceived risk had significant negative influence on BI to use VAT. Self-efficacy had significantly influenced perceived enjoyment, accounting for 35.5% of the variance of perceived enjoyment. This investigation reveals that performance efficiency and user enjoyment are key determinants in dentists’ decision to adopt VAT. Concerns regarding the privacy of VAT also play a crucial role in its acceptance. This study represents the first documented inquiry into dentists’ reception of VAT, laying groundwork for future research and implementation strategies.

## Author summary

Voice assistants, such as Siri and Alexa, have become more common in daily life, and this technology could provide increased efficiency in the dental field. Voice assistants could allow dentists to engage with computers and navigate a patient’s record via voice interaction. Since dentists are often utilizing their hands during patient care, this technology would increase productivity and hygiene during clinical practice and operations. In this research study, we aimed to investigate dentists’ perception in utilizing voice assistants in the clinic and to understand what factors influence their perception to adopt this technology. Dentists were invited to participate in an online survey. Survey findings reported that dentists perceive that voice assistants could improve their daily performance and the enjoyment of using a new technology could affect their perception. Security and privacy issues could discourage dentists from adopting voice assistants in the clinics. This research provides a foundation for features to design and implement voice assistants for the dental office.

## Introduction

Computer utilization in the dental office has experienced dramatic growth over the past 40 years [1,2]. With an exponential growth in the early 2000s, it reached from 24.6% of dentists using a computer chairside in their offices (2006) [3] to 85% in 2009 [4]. One of the most important enhancements to computers in dental practice is the implementation of the electronic health record (EHR). EHRs allow enhanced patient safety and outcomes by providing more-comprehensive patient records, easier transfer of health information to other medical providers, and reduction of paper use [5,6,7].

Dentists, dental hygienists, and dental assistants often interact with the EHR through mouse and keyboard, however there are several infection control and efficiency considerations that make this type of input problematic. Touching the mouse and keyboard while performing clinical care creates infection control concerns, while also leading to increased costs and waste with barrier devices [8,9,10]. Dental providers rely on their hands during procedures and examinations, and the computer is often out of reach or in a position that is not ideal for input [8,11]. Time spent using the EHR can also affect patients directly, with a previous study citing 58.8% of patients finding a clinician’s computer use distracting, even when used for patient care, and 69% stating they felt frustrated when the dentist was using the computer [6]. Due to the inconvenient positioning and concerns with infection control when using the mouse and keyboard while examining patients, dentists often delegate EHR input to dental auxiliaries [12]; this introduces an error opportunity due to miscommunication. Clinicians also communicate information in an unstructured manner, which then has to be entered in a structured form in the chart [13]. Previous research has shown that clinical systems are more accurate when clinicians directly enter the data [14]. There is opportunity for improved methods for dental providers to interact with the computer and EHR during patient care.

Voice interaction and input incorporated into dental charting may mitigate many of the problems associated with mouse and keyboard. Previous research has shown that dentists desire voice interactive software [3,15]. Dental speech applications are currently available for EHRs, however there are limitations [8]. Most of these software programs currently utilize two distinct types of voice input: command-driven and dictation [16,17]. The command-driven software allows users to navigate the EHR and enter information [12]; however, this mode of input requires memorization of specific words and prompts to navigate and enter information into the EHR, limiting its overall usefulness [11,12]. The voice dictation feature allows direct transcription of spoken words into the EHR [13]. An earlier study reported that 13% of dentists were using one of these modalities of voice interactive software, and another 16% of dentists had tried voice interaction, but discontinued use of the software [3]. The common reasons cited for discontinuation were errors in speech recognition and inefficiencies [3].

One of the problems with previous implementations of voice interactive software is that they do not allow natural language communication with the EHR, except for dictation tools. In addition, speech recognition, automatic speech recognition (ASR) in this software historically underperform [12]. With the recent improvements in Artificial Intelligence (AI), ASR and natural language processing (NLP) algorithms would allow the use of natural communication and vocabulary to navigate and enter information into the EHR [12,18]. For example, a dental practitioner could say, “there is mesial caries on #3,” and the computer would be able to interpret that input and place a marked carious lesion on that tooth at the appropriate location on the odontogram. The dental professional could also say, “#3 has mesial caries,” with the same result. NLP and machine learning algorithms help to process unstructured data, such as voice input transcripts, and change it to structured data, such as dental charting within an EHR [13]. Voice interaction would also allow navigation of the dental chart [16]. For example, the dental practitioner could say, “open the latest radiographs for this patient,” and the computer would be capable of bringing up the relevant radiographs on the patient. With the application of ASR and NLP with voice assistant technologies enabling natural communication with devices, aforementioned issues on hygiene, auxiliary devices, and recall is addressable.

Despite the benefits of voice assistant technology (VAT) listed above, the development of modern technology is not beneficial if practitioners do not perceive a value to use it. User acceptance is an important factor in determining if a new technology will be implemented [19–21]. With novel software and equipment, it is essential to understand the psychological and social factors affecting its implementation [19,22]. The primary objective of this study was to assess dentists’ perceptions and intention to use VAT. A secondary objective was to understand which factors are important in the adoption of VAT.

## Theoretical framework

A number of conceptual and theory-driven models measuring technology acceptance have been developed, validated and tested in the literature [23]. One of the most-utilized models is the Unified Theory of Acceptance and Use of Technology (UTAUT), developed by Venkatesh et al [24]. UTAUT is a combination of 8 behavioral assessment models (including Theory of Reasoned Action, Technology Acceptance Model, Theory of Planned Behavior, Innovation of Diffusion Theory and Social Cognitive Theory) towards explaining a user’s intention to use a technology. This instrument has proven to explain 69% of intention-to-use a technology [24,25]. An extension of the UTAUT model was developed by Chao, which is an extended UTAUT model to include trust, perceived risk, self-efficacy, satisfaction, and perceived enjoyment constructs [20]. This model has the benefit of including trust and perceived risk, which are found to be important when measuring voice interactive technology. The UTAUT model has been widely used in many different fields, including dentistry, towards assessing user perceptions and intention to use technology in healthcare delivery [19,26–29].

## Materials and methods

### Recruitment

Licensed dentists in the state of Ohio were recruited. With the support from the Ohio State Dental Board, the invitation to participate was sent to 7,545 dentists via email in December 2021, with a reminder email in January 2022. The email contained a brief description of the survey, and a link to participate [S1 Appendix] which redirected the participant to a REDCap (Vanderbilt University, Nashville, Tennessee) questionnaire. The questionnaire included consent for participation [S2 Appendix], a demographic questionnaire [S3 Appendix], and the survey [S4 Appendix]. Participation was voluntary, and no incentive or reimbursement was provided to participants. Subjects were able to complete the survey at any time during the period the survey was open for completion without a time limitation. The survey was open for completion by each subject one time only. This study was approved by the Institutional Review Board of Nationwide Children’s Hospital, Columbus, Ohio (STUDY00000418).

### Research model

**Fig 1:**
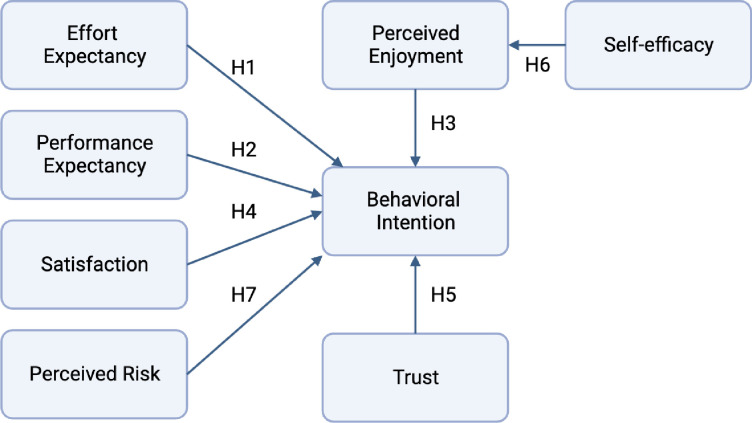
Proposed research model.

Our study is informed by an extended UTAUT model which includes the constructs of Trust, Self-Efficacy, Perceived Risk, Satisfaction, and Perceived Enjoyment [20] in addition to original UTAUT constructs of Performance Expectancy (PE), Effort Expectancy (EE), Social Influence (SI), Facilitating Conditions (FC), and Behavioral Intention (BI) [24]. The Extended UTAUT model was selected due to the added value of additional constructs to assess multiple aspects of dentist behaviors. We refined the extended UTAUT model in our study to focus on the behavioral intention to use of the proposed technology (Fig 1). Using a simplified relationship on our research model allowed us to investigate the direct effects of the constructs on BI.

In our research model (Fig 1) PE was one of the original UTAUT constructs, and it was found in previous studies to be the most important factor in behavioral intention to use a new technology [20,24]. PE is described as “the degree to which an individual believes that the system helps improve job performance” [20,24]. EE is another construct of the original UTAUT which was also used in this extended model. EE is defined as “the degree of ease associated with the use of the system” [20,24]. BI is the main dependent variable that the UTAUT model attempts to measure in this study. BI is “the degree to which a person has formulated conscious plans regarding whether to perform a specified behavior” [20,24]. Satisfaction with a software or system can be a major influence on BI [20,30]. Satisfaction has previously been defined as a “users’ level of satisfaction with reports, websites, and support services” [20,30]. Although the influence of trust on BI is inconclusive from previous studies, the authors believed that trust was a major factor in the specific case of voice-listening software [20,29,31–33]. Trust measures the degree of reliability and perceived truthfulness of a software [20,31]. Perceived enjoyment of using the voice assistant technology serves to add an emotional component to the survey [20]. Perceived enjoyment is “the extent to which the activity of using a specific system is perceived to be enjoyable in its own right, aside from any performance consequences resulting from system use” [20,34]. Self-efficacy refers to an individual’s views on their ability to perform a task sufficiently [20,35]. Like trust, perceived risk is a key factor in measuring technology which may produce concerns about privacy and security. The greater the perceived risk of software, such as program errors, privacy concerns, or incompatibility, the less likely the software will be adopted [20,36,37]. Perceived risk is the “potential for loss in the pursuit of a desired outcome using an e-service” [20,38].

Our proposed hypotheses in association with our research model are listed below:

H1: EE has a significant influence on BI to use voice assistant technology.
H2: PE has a significant influence on BI to use voice assistant technology.
H3: Perceived enjoyment has a significant influence on BI to use voice assistant technology.
H4: Satisfaction has a significant influence on BI to use voice assistant technology.
H5: Trust has a significant influence on BI to use voice assistant technology.
H6: Self-efficacy has a significant influence on perceived enjoyment of using voice assistant technology.
H7: Perceived risk has a significant influence on BI to use voice assistant technology.

### Survey instrument

The extended UTAUT model by Chao et al.[20] was used as the instrument in this survey, and it consisted of 31 questions [S4 Appendix]. A 5-point Likert scale was used to collect responses, ranging from strongly disagree (1) to strongly agree (5). The survey questions were kept in their original form to maintain integrity with a slight change (the technology name changed to “voice assistant technology”).

### Data analysis

The responses from the survey were exported from REDCap to Microsoft Excel sheets for analysis. Statistical R version 3.6.2 (R Foundation for Statistical Computing, Vienna, Austria) was used to analyze the data.

#### Measurement model evaluation

Prior to analyzing the relationships between constructs, it was necessary to evaluate the measurement models to ensure that the items accurately represented the constructs. Constructs that did not meet validation criteria were excluded from the model. This evaluation focused on assessing the reliability and validity of the measurements to identify the constructs relevant for the model [39].

Internal reliability was assessed using Cronbach’s alpha (α) and composite reliability (CR), while validity was evaluated through convergent validity (CV) and discriminant validity (DV). Convergent validity was determined by the average variance extracted (AVE), and discriminant validity was calculated using the square root of AVE. Additionally, item loadings were analyzed to decide the inclusion or exclusion of constructs in the model. Constructs with non-significant loadings were removed due to their unreliability in the model [40].

#### Structural model and hypotheses testing

Structural model measurements were completed with partial least squares regression (PLSR). PLSR is a structural equation modeling (SEM) technique to determine the validity and reliability of both the structure and the measurements of a model through bootstrapping of the path [20]. PLSR utilizes bootstrapping to determine the strength of the path coefficients [20]. PLSR is especially useful in structural equation modeling when a model is still in the theoretical stages and has not been tested completely [20,40].

We used PLSR to analyze the path relationships among constructs in our research model and to identify the coefficient of determination (R^2^). A path relationship value of P<0.05 was determined to be significant.

#### Power analysis

A power analysis was conducted to determine the number of responses needed in order to have adequate data for statistical analysis. We found a minimum of 60 participants, or at least 10 times the highest number of construct paths directed at the latent variable (BI), would be needed for analysis [39]. The authors aimed to receive 200–300 completed surveys, which would be adequate for analysis in the PLSR-SEM employed in this study [41].

## Results

### Demographics

The results of the demographic questionnaire are shown in Table 1. Of the 7,545 surveys sent, 257 were completed with a response rate of 3.4%. The average age of the sample was 51 (range 26–82). The majority were white (86.4%) and male (59.1%). Over half of the dentists who completed the survey had practiced for longer than 20 years. The largest specialty group was general dentists (64.6%), with pediatric dentists making up the second largest group (14%). Dentists who practiced in the suburbs (62.3%) and those in urban settings (20.6%) constituted the majority of the respondents. Over half of the participants have not treated patients on Medicaid. Only 14.4% stated that they had never used VAT, and 20.6% stated that they used VAT regularly in their daily lives.

**Table 1:** Demographics.

Items	N (257)	%
**Age**		
Mean (Standard Deviation)	51 (14.3)	
Min-Max	26–82	
**Gender**		
Female	102	39.7%
Male	152	59.1%
Prefer not to answer	3	1.2%
**Race/Ethnicity**		
Asian	14	5.4%
Black or African American	5	1.9%
Native Hawaiian or Other Pacific Islander	1	0.4%
White	222	86.4%
Multi-race	6	2.3%
Other	9	3.5%
**Familiarity with voice assistant technology**		
Never used	37	14.4%
Used a few times	92	35.8%
Use often	75	29.2%
Use everyday	53	20.6%
**Time since completion of dental education**		
< 5 years ago	35	13.6%
5–10 years ago	36	14.0%
11–15 years ago	23	8.9%
16–20 years ago	19	7.4%
>20 years ago	144	56.0%
**Specialty**		
General Dentist	166	64.6%
Pediatric Dentist	36	14.0%
Orthodontist	15	5.8%
Endodontist	12	4.7%
Periodontist	4	1.6%
Oral and Maxillofacial Surgeon	11	4.3%
Prosthodontist	4	1.6%
Dental Anesthesiologist	3	1.2%
Dental Public Health	3	1.2%
Other	3	1.2%
**What is the location of your practice?**		
Rural	44	17.1%
Suburban	160	62.3%
Urban	53	20.6%
**Estimate percentage of patient population with Medicaid/Medicaid HMO**		
0%	147	57.2%
1–24%	56	21.8%
25–50%	18	7.0%
more than 50%	36	14.0%

### Measurement model evaluation

Table 2 reports construct reliability results. The item loading values ranged between 0.590–0.928, which was found acceptable with above 0.4 [39]. Cronbach’s alpha values were between 0.79–0.93 with above the acceptable value of 0.7 [42]. The AVE, which measures the CV, ranged from 0.587 to 0.795, with all constructs meeting the requirement of above 0.5 [43]. All values of composite reliability were satisfactory with above 0.7 [39].

**Table 2:** Construct reliability.

Construct	No. of items	Item loading	Cronbach’s α	AVE	CR
Self-efficacy (SE)	3	0.834–0.845	0.79	0.704	0.878
Perceived Enjoyment (PEN)	3	0.866–0.919	0.87	0.793	0.920
Perceived Risk (PR)	3	0.708–0.875	0.76	0.610	0.864
Effort Expectancy (EE)	5	0.770–0.860	0.88	0.672	0.912
Performance Expectancy (PE)	4	0.853–0.928	0.93	0.820	0.948
Satisfaction (SAT)	5	0.786–0.914	0.91	0.729	0.931
Trust (TRU)	5	0.590–0.878	0.82	0.587	0.877
Behavioral Intention (BI)	3	0.866–0.916	0.87	0.795	0.921
AVE: Average Variance Extracted; CR: Composite Reliability (also known as Dillon-Goldstein’s rho).					

Table 3 shows correlation matrix and square root of AVE. The DV for each construct met the requirement of the intra-construct (bolded diagonal values) as the square root of AVE was greater than the construct correlation values. In other terms, the questions within each construct correlated more heavily with each other than the correlation between two separate constructs [39].

**Table 3:** Correlation matrix and square root of AVE.

Construct	Mean	SD	SE	PEN	PR	EE	PE	SAT	TRU	BI
Self-efficacy (SE)	3.63	0.75	**0.840** *							
Perceived Enjoyment (PEN)	3.40	0.76	0.596*	**0.890** *						
Perceived Risk (PR)	2.81	0.80	-0.307*	-0.384*	**0.780** *					
Effort Expectancy (EE)	3.64	0.69	0.811*	0.684*	-0.250*	**0.820** *				
Performance Expectancy (PE)	3.40	0.76	0.490*	0.704*	-0.467*	0.527*	**0.910** *			
Satisfaction (SAT)	3.34	0.76	0.670*	0.778*	-0.420*	0.754*	0.640*	**0.850** *		
Trust (TRU)	3.22	0.66	0.612*	0.619*	-0.604*	0.565*	0.651*	0.715*	**0.770** *	
Behavioral Intention (BI)	3.81	0.83	0.566*	0.775*	-0.511*	0.598*	0.782*	0.705*	0.625*	**0.890** *

SD, Standard deviation; Bolded values on the diagonal are the square root of the AVE.

Values on the off-diagonal represent inter-construct correlations.

*P < 0.05

### Structural model and hypotheses testing

**Fig 2:**
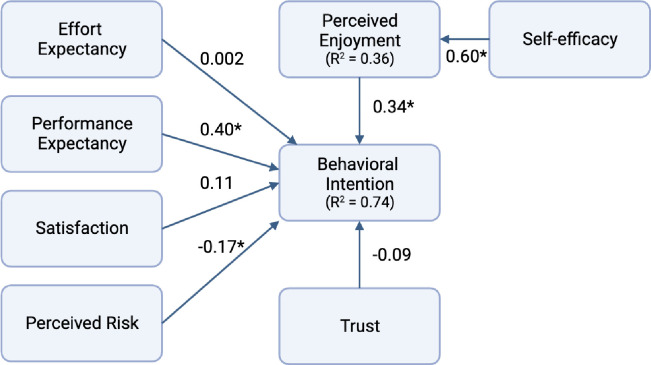
Path coefficients for the research model. Value on path: standardized coefficients (β), R^2^: Coefficient of determination and *p < 0.05.

The structural model analysis showed that perceived enjoyment and PE had a significant positive effect on BI to use VAT. Perceived risk had a significant negative effect on BI to use VAT. Self-efficacy had a significant positive effect on perceived enjoyment. The model and direct effects of each construct are shown in Fig 2. Hypotheses 2, 3, 6, and 7 were supported in this model. Trust, satisfaction, and EE did not have a significant influence on BI to use VAT. The values of the construct’s effects, P-Values, and support for each hypothesis are shown in Table 4. Self-efficacy accounted for 35.5% of the variance in perceived enjoyment. This model accounted for 74.2% of the variance in BI to use VAT [39].

**Table 4:** Hypothesis testing.

Hypothesis	Relation	Direct Effect	Indirect Effect	Total Effect	P Value	Status
H6	SE -> PEN	0.60	0.00	0.60	<0.0001	Supported
H3	PEN -> BI	0.33	0.00	0.33	<0.0001	Supported
H1	EE -> BI	0.002	0.00	0.002	0.98	Not supported
H2	PE -> BI	0.40	0.00	0.40	<0.0001	Supported
H4	SAT -> BI	0.11	0.00	0.11	0.09	Not supported
H5	TRU -> BI	-0.09	0.00	-0.09	0.13	Not supported
H7	PR -> BI	-0.17	0.00	-0.17	<0.0001	Supported

## Discussion

VAT can allow natural speech communication, navigation of a system, and information acquisition in a hands-free manner in a clinical setting [44]. Our study reported the factors influencing dentist’s intention to use VAT at clinic. The findings provide insight about dentist perceptions that can be used as input during VAT development and implementation while focusing on efficiency, reducing errors, and increasing hygiene during dental appointments through natural language interaction, allowing navigation and data input in a hands-free manner.

Our model accounted for 74.2% of the variance in BI to use VAT. In other words, the constructs of this model captured 74.2% of the factors which are important in a dentists’ decision to use VAT. This informs literature and fills the gap with a satisfactory level of variance explained, compared to prior studies with the UTAUT model [24] and other behavioral models to explain medical technology use [45].

Trust was found to have a negative affect but not significant influence on BI. In daily life use, 41% of VAT users have concerns and trust issues due to VAT’s passive listening ability and compromised privacy [46]. Trust has been shown to be a significant factor in technology adoption in general as well [20,33,47,48]. One reason could be that practitioners’ current trust in medical systems being used in a clinical setting, given the previous experiences with dictation tools used in clinical settings. Therefore, they considered VAT to be part of clinical tools they have already established knowledge and also having mixed thoughts about trust [49].

EE was one of the original constructs of the UTAUT model [24], but it was not found to have a significant influence on BI. The effort spent in learning to use a technology has been shown to be an influencing factor on its adoption [20,27]. With around 4.2 billion devices utilizing VAT around the world, the familiarity with VAT may have decreased the effort expected to utilize in the dental office [50]. Also, nearly 50% of the participants of the survey used VAT daily or very often also likely decreased the expected effort of using this technology.

In the path analysis, PE had the highest influence on behavioral intention to use VAT. This finding is consistent with previous literature on technology use [20,27–29,51]. It is not surprising that PE was the most important factor in this model as previous research has shown that one of the most common reasons for abandonment of the current command-driven voice software among dentists was inefficient data entry and errors in speech recognition [3]. VAT would eliminate some of the difficulties with the inefficiencies of command-driven software by providing a more engaging platform between the dentist and the EHR through natural conversation.

Perceived enjoyment had a significant influence on BI. The direct association between perceived enjoyment and BI is novel to this study. The questions within this construct were reflective of the dentists’ enjoyment in using VAT outside of a dental context. Previous literature focused on improved performance and efficiency when using VAT over standard mouse and keyboard for data input into the EHR, however, we observed that perceived enjoyment of using the VAT had an almost equal influence on BI as performance expectancy in this model [16]. Although satisfaction is a similar construct to perceived enjoyment and has been shown to be important in technology acceptance, satisfaction did not have a significant influence in this population [20,30]. Another important insight gained was the significant effect of self-efficacy on perceived enjoyment. Enjoyment of technology has been shown to be influenced by proficiency in using the technology [20]. VAT was shown to be a familiar concept among those surveyed in this study, with nearly 50% using VAT often or daily. With this level of familiarity with VAT, it is likely that dentists have increased enjoyment informed by their self-efficacy with using this technology. However, this finding also indicates that it is important to focus on improving users’ self-efficacy, via training and education modules, for a successful transition to use VAT in clinics [52].

Similar to trust, perceived risk has a negative but significant relationship with BI, which measures the importance of privacy in VAT as a clinical technology. Unlike trust, risk may have influenced a more perceived negative image towards the technology. The findings suggest that the risk of using VAT may be a deterrent in using this technology in the dental setting. [53] X. Given the fact that VAT can be compromised [54,55] and may result in loss of protected health information, it may cause dentists to be held accountable [56]. Adoption of VAT in dental offices will be influenced by the security risks of use.

### Practical implications

There are a number of practical implications of this study. The positive perception of VAT in terms of performance and enjoyment among dentists suggests that its integration could enhance operational efficiency and workplace satisfaction. This finding indicates a potential for increased adoption, particularly if the technology is tailored to meet the specific needs of dental practices. In addition, developers and implementers need to consider building trust, privacy and low risk systems to gain wider acceptance within the dentistry. Furthermore, the varying levels of technological comfort among dentists call for targeted training and support programs to ensure effective utilization of VAT. Finally, the insights from this study could guide policymakers and technology developers in shaping strategies for technology integration in healthcare settings, specifically in areas with similar characteristics to the target population.

### Limitations

We recruited via a convenience sampling which limited study samples to licensed dentists in the state of Ohio. Therefore, the participants of this study may not be reflective of dentists in other states and regions of the United States or other parts of the world, which limits generalizability of the study results. Another limitation was the low response rate in this survey. The response rate was 3.4%, which is lower than the average health care response rate of 53% mentioned in previous research [57]. Although this study met the minimum number of participants needed for statistical analysis, the low response rate suggests the generalizability of results might be affected by bias, particularly from response bias [58]. Although it was not possible to measure (since no personal information was collected from the participants), there could be more personal and demographic factors which may have contributed to a response bias. Since the survey was only sent through email, those who took the survey may have been more comfortable with using technology. It is also feasible to postulate that at least some of the responders were already interested in VAT, and those who were interested represented a disproportionate amount of the sample which completed the survey. This bias may have skewed the results more in creating positive bias in responses. In addition, the absence of a non-responder analysis and qualitative data might limit the depth and context of findings. Recall bias could also affect results if participants were required to remember past experiences. Finally, the cross-sectional nature of the study restricts causal inferences and understanding changes over time.

### Future research

There has been increased developments in VAT with large technology companies, such as Apple, Microsoft, and Google [59]. However, VAT usually remains as a common consumer product, but a comprehensive system has not yet been studied in dentistry. Therefore, future implementation research is necessary at the clinical setting. To improve diversity and sample size in responses, future research is planned on acceptance of VAT. Conducting a survey on VAT with a broader pool of dentists will provide more foundational knowledge on the interest and important factors in adopting and using VAT. Using influencing factors revealed in this study, pilot testing of a developed VAT in a controlled setting, such as a simulation center, could be used to further test the benefits and drawbacks of this technology for dentistry. Building on this study’s findings, we could include the acceptance of other artificial AI systems by practitioners in future studies [17,60,61]. In addition, we plan to investigate the acceptance of both VAT and AI by patients and caregivers. This could give insight on both the practitioner and patient perspectives on these emerging technologies.

## Conclusion

VAT may play a role in the dental office in the future, as dentists showed interest in using this technology. The ability to use VAT effectively has a high influence on factors influencing dentists’ intention. The performance, risk and enjoyment of using this technology are found to be important components in the development and implementation among dentists.

## Supporting information

S1 Appendix Invitation Email to Participate in Study. (DOCX)

S2 Appendix Consent Form. (DOCX)

S3 Appendix Demographic Questionnaire. (DOCX)

S4 Appendix Survey. (DOCX)

## References

[1] Schwei KM, Cooper R, Mahnke AN, Ye Z, Acharya A. Exploring dental providers’ workflow in an electronic dental record environment. Appl Clin Inform. 2016;7(2):516–533. doi: 10.4338/ACI-2015-11-RA-0150 27437058 PMC4941857

[2] Acharya A, Schroeder D, Schwei K, Chyou PH. Update on electronic dental record and clinical computing adoption among dental practices in the United States. Clin Med Res. 2017;15(3–4):59–74. doi: 10.3121/cmr.2017.1380 29229631 PMC5849439

[3] Schleyer TKL, Thyvalikakath TP, Spallek H, Torres-Urquidy MH, Hernandez P, Yuhaniak J. Clinical Computing in General Dentistry. J Am Med Inform Assoc. 2006;13(3):344–352. doi: 10.1197/jamia.M1990 16501177 PMC1513654

[4] American Dental Association. Survey of current issues in dentistry: dentists’ computer use. Chicago: American Dental Association; 2001.

[5] HealthIT.gov. Benefits of EHRs. Improved diagnostics & patient outcomes. [updated 2014 March 19; accessed 2022 Jan 10]. Available from: https://www.healthit.gov/providers-professionals/improved-diagnostics-patient-outcomes.

[6] Asan O, Ye Z, Acharya A. Dental care providers’ and patients’ perceptions of the effect of health information technology in the dental care setting. J Am Dent Assoc. 2013;144(9):1022–1029. doi: 10.14219/jada.archive.2013.0229 23989841

[7] Jabali AK, Waris A, Khan DI, Ahmed S, Hourani RJ. Electronic health records: Three decades of bibliometric research productivity analysis and some insights. Inform Med Unlocked. 2022;29. doi: 10.1016/j.imu.2022.100872

[8] Irwin JY, Wali T, Fernando S, Schleyer T. Speech Recognition in Dental Software Systems: Features and Functionality. AMIA Annu Symp Proc. 2007;987.18694087

[9] Keesara S, Jonas A, Schulman K. Covid-19 and Health Care’s Digital Revolution. N Engl J Med. 2020;382(23):1–3. doi: 10.1056/NEJMp2005835 32240581

[10] Sezgin E, Huang Y, Ramtekkar U, Lin S. Readiness for voice assistants to support healthcare delivery during a health crisis and pandemic. Npj Digit Med. 2020;3(1). doi: 10.1038/s41746-020-00332-0 33015374 PMC7494948

[11] Vishwanathaiah S, Fageeh HN, Khanagar SB, Maganur PC. Artificial Intelligence Its Uses and Application in Pediatric Dentistry: A Review. Biomedicines. 2023;11(3):788. doi: 10.3390/biomedicines11030788 36979767 PMC10044793

[12] Irwin RY. Speech to Chart: Speech Recognition and Natural Language Processing for Dental Charting. University of Pittsburgh; 2009. doi: 10.1088/1751-8113/44/8/085201

[13] Kreimeyer K, Foster M, Pandey A, Arya N, Halford G, Jones SF, et al. Natural language processing systems for capturing and standardizing unstructured clinical information: A systematic review. J Biomed Inform. 2017;73:14–29. doi: 10.1016/j.jbi.2017.07.012 28729030 PMC6864736

[14] Doolan DF, Bates DW. Computerized physician order entry systems in hospitals: mandates and incentives. Health Aff (Millwood). 2002 Jul-Aug;21(4):180–8. doi: 10.1377/hlthaff.21.4.180 12117128

[15] Agrawal P, Nikhade P. Artificial Intelligence in Dentistry: Past, Present, and Future. Cureus. 2022;14(7). doi: 10.7759/cureus.27405 36046326 PMC9418762

[16] Drevenstedt GL, McDonald JC, Drevenstedt LW. The role of voice-activated technology in today’s dental practice. J Am Dent Assoc. 2005;136(2):157–161. doi: 10.14219/jada.archive.2005.0135 15782518

[17] Zhang J, Wu J, Qiu Y, Song A, Li W, Li X, Liu Y. Intelligent speech technologies for transcription, disease diagnosis, and medical equipment interactive control in smart hospitals: A review. Comput Biol Med. 2023;153(Dec 2022):106517. doi: 10.1016/j.compbiomed.2022.106517 36623438 PMC9814440

[18] Sezgin E, D’Arcy S. Editorial: Voice Technology and Conversational Agents in Health Care Delivery. Front Public Health. 2022 May;10:1–3. doi: 10.3389/fpubh.2022.887492 35712270 PMC9196328

[19] Alabdullah JH, Van Lunen BL, Claiborne DM, Daniel SJ, Yen CJ, Gustin TS. Application of the unified theory of acceptance and use of technology model to predict dental students’ behavioral intention to use teledentistry. J Dent Educ. 2020;84(11):1262–1269. doi: 10.1002/jdd.12304 32705688

[20] Chao CM. Factors determining the behavioral intention to use mobile learning: An application and extension of the UTAUT model. Front Psychol. 2019 Jul;10:1–14. doi: 10.3389/fpsyg.2019.01652 31379679 PMC6646805

[21] Davis FD, Bagozzi RP, Warshaw PR. User acceptance of computer technology: a comparison of two theoretical models. Manag Sci. 1989;35(8):982–1003. doi: 10.1287/mnsc.35.8.982

[22] Hu PJ, Chau PYK, Liu Sheng OR, Tam KY. Examining the Technology Acceptance Model Using Physician Acceptance of Telemedicine Technology. J Manage Inf Syst. 1999;16(2):91–112. doi: 10.1080/07421222.1999.11518247

[23] Holden RJ, Karsh BT. The Technology Acceptance Model: Its past and its future in health care. J Biomed Inform. 2010;43(1):159–172. doi: 10.1016/j.jbi.2009.07.002 19615467 PMC2814963

[24] Venkatesh V. USER ACCEPTANCE OF INFORMATION TECHNOLOGY: TOWARD A UNIFIED VIEW. MIS Q. 2003;27(3):425–478. doi: 10.1103/PhysRevB.57.R14040

[25] Kijsanayotin B, Pannarunothai S, Speedie SM. Factors influencing health information technology adoption in Thailand’s community health centers: Applying the UTAUT model. Int J Med Inform. 2009;78(6):404–416. doi: 10.1016/j.ijmedinf.2008.12.005 19196548

[26] Cimperman M, Brenčič MM, Trkman P. Analyzing older users’ home telehealth services acceptance behavior—applying an extended UTAUT model. Int J Med Inform. 2016;90:22–31. doi: 10.1016/j.ijmedinf.2016.03.002 27103194

[27] Šumak B, Šorgo A. The acceptance and use of interactive whiteboards among teachers: differences in UTAUT determinants between pre- and post-adopters. Comput Hum Behav. 2016;64:602–620. doi: 10.1016/j.chb.2016.07.037

[28] Hoque R, Sorwar G. Understanding factors influencing the adoption of mHealth by the elderly: an extension of the UTAUT model. Int J Med Inform. 2017;101:75–84. doi: 10.1016/j.ijmedinf.2017.02.002 28347450

[29] Khalilzadeh J, Ozturk AB, Bilgihan A. Security-related factors in extended UTAUT model for NFC based mobile payment in the restaurant industry. Comput Hum Behav. 2017;70:460–474. doi: 10.1016/j.chb.2017.01.001

[30] DeLone WH, McLean ER. Information systems success measurement. Found Trends Inf Syst. 2016;2:1–116. doi: 10.1561/2900000005

[31] Understanding Arpaci I. and predicting students’ intention to use mobile cloud storage services. Comput Hum Behav. 2016;58:150–157. doi: 10.1016/j.chb.2015.12.067

[32] Alalwan AA, Dwivedi YK, Rana NP. Factors influencing adoption of mobile banking by jordanian bank customers: extending UTAUT2 with trust. Int J Inf Manage. 2017;37:99–110. doi: 10.1016/j.ijinfomgt.2017.01.002

[33] Kabra G, Ramesh A, Akhtar P, Dash MK. Understanding behavioural intention to use information technology: insights from humanitarian practitioners. Telematics Inform. 2017;34:1250–1261. doi: 10.1016/j.tele.2017.05.010

[34] Park Y, Son H, Kim C. Investigating the determinants of construction professionals’ acceptance of web-based training: an extension of the technology acceptance model. Autom Constr. 2012;22:377–386. doi: 10.1016/j.autcon.2011.09.016

[35] Bandura A. Social Foundations of Thought and Action: A Social Cognitive Theory. Englewood Cliffs, NJ: Prentice-Hall; 1986.

[36] Hanafizadeh P, Behboudi M, Koshksaray AA, Tabar MJS. Mobile-banking adoption by Iranian bank clients. Telematics Inform. 2014;31:62–78. doi: 10.1016/j.tele.2012

[37] Alsyoud A, Lutfi A, Alsubahi N, Alhazmi FN, Al-Mugheed K, Anshasi RJ, et al. The Use of a Technology Acceptance Model (TAM) to Predict Patients’ Usage of a Personal Health Record System: The Role of Security, Privacy, and Usability. Int J Environ Res Public Health. 2023;20(2). doi: 10.3390/ijerph20021347 36674105 PMC9859518

[38] Featherman MS, Pavlou PA. Predicting e-services adoption: a perceived risk facets perspective. Int J Hum Comput Stud. 2003;59:451–474. doi: 10.1016/s1071-5819(03)00111-3

[39] Hair JF, Ringle CM, Sarstedt M. PLS-SEM: Indeed a Silver Bullet. J Market Theory Pract. 2011;19(2):139–152. doi: 10.2753/MTP1069-6679190202

[40] Nunnally JC. Psychometric Theory (2nd ed). New York: McGraw-Hill; 1978.

[41] Hair J, Hult GTM, Ringle CM, Sarstedt M. A primer on partial least squares structural equation modeling (PLS-SEM) (2nd ed). SAGE Publications; 2016.

[42] Hair F Jr, Black WC, Babin BJ, Anderson RE. Multivariate Data Analysis: A Global Perspective, 7th Ed. New York, NY: MacMillan; 2010.

[43] Segars AH. Assessing the unidimensionality of measurement: a paradigm and illustration within the context of information systems research. Omega. 1997;25(1):107–121. doi: 10.1016/S0305-0483(96)00051-5

[44] Kumah-Crystal YA, Pirtle CJ, Whyte HM, Goode ES, Anders SH, Lehmann CU. Electronic Health Record Interactions through Voice: A Review. Appl Clin Inform. 2018;9(3):541–552. doi: 10.1055/s-0038-1666844 30040113 PMC6051768

[45] Sezgin E, Yıldırım SÖ. A Literature Review on Attitudes of Health Professionals towards Health Information Systems: From e-Health to m-Health. Procedia Technol. 2014;16:1317–1326. doi: 10.1016/j.protcy.2014.10.148

[46] Olson C. New report tackles tough questions on voice and AI. [Internet]. Microsoft Ads Blog. 2019 Apr. Available from: https://about.ads.microsoft.com/en-us/blog/post/april-2019/new-report-tackles-tough-questions-on-voice-and-ai

[47] Koufaris M, Hampton-Sosa W. The Development of Initial Trust in an Online Company by New Customers. Inf Manage. 2004;41:377–397. doi: 10.1016/j.im.2003.08.004

[48] May A, Mitchell V, Piper J. A user-centred design evaluation of the potential benefits of advanced wireless sensor networks for fire-in-tunnel emergency response. Fire Saf J. 2014;63:79–88. doi: 10.1016/j.firesaf.2013.11.007

[49] Rathert C, Porter TH, Mittler JN, Fleig-Palmer M. Seven years after Meaningful Use: Physicians’ and nurses’ experiences with electronic health records. Health Care Manage Rev. 2019 Jan/Mar;44(1):30–40. doi: 10.1097/HMR.0000000000000168 28614166

[50] Statista. Number of digital voice assistants in use worldwide from 2019 to 2024. [Internet]. 2020. Available from: https://www.statista.com/statistics/973815/worldwide-digital-voice-assistant-in-use/

[51] Šumak B, Pušnik M, Heričko M, Šorgo A. Differences between prospective, existing, and former users of interactive whiteboards on external factors affecting their adoption, usage, and abandonment. Comput Hum Behav. 2017;72:733–756. doi: 10.1016/j.chb.2016.09.006

[52] Rahman MS, Ko M, Warren J, Carpenter D. Healthcare Technology Self-Efficacy (HTSE) and its influence on individual attitude: An empirical study. Comput Hum Behav. 2016;58:12–24. doi: 10.1016/j.chb.2015.12.016

[53] Bolton T, Dargahi T, Belguith S, Al-Rakhami MS, Sodhro AH. On the security and privacy challenges of virtual assistants. Sensors. 2021;21(7). doi: 10.3390/s21072312 33810212 PMC8036736

[54] Mitev R, Miettinen M, Sadeghi AR. Alexa lied to Me: Skill-based man-in-the-middle attacks on virtual assistants. AsiaCCS 2019—Proceedings of the 2019 ACM Asia Conference on Computer and Communications Security. 2019 Jul;465–478. doi: 10.1145/3321705.3329842

[55] Zhang N, Mi X, Feng X, Wang X, Tian Y, Qian F. Dangerous skills: Understanding and mitigating security risks of voice-controlled third-party functions on virtual personal assistant systems. Proc IEEE Symp Secur Priv. 2019 May;1381–1396. doi: 10.1109/SP.2019.00016

[56] Kessler SR, Pindek S, Kleinman G, Andel SA, Spector PE. Information security climate and the assessment of information security risk among healthcare employees. Health Inform J. 2020;26(1):461–473. doi: 10.1177/1460458219832048 30866704

[57] Cho YI, Johnson TP, VanGeest JB. Enhancing Surveys of Health Care Professionals: A Meta-Analysis of Techniques to Improve Response. Eval Health Prof. 2013;36(3):382–407. doi: 10.1177/0163278713496425 23975761

[58] Funkhouser E, Vellala K, Baltuck C, Cacciato R, Durand E, McEdward D, et al. Survey Methods to Optimize Response Rate in the National Dental Practice–Based Research Network. Eval Health Prof. 2017;40(3):332–358. doi: 10.1177/0163278715625738 26755526 PMC5002250

[59] Hoy MB. Alexa, Siri, Cortana, and more: an introduction to voice assistants. Med Ref Serv Q. 2018 Jan 2;37(1):81–88. doi: 10.1080/02763869.2018.1404391 29327988

[60] Hain P, Cancio P, Morales G, Nhieu M, Antonio RG, Moreno JV. Improving Nurse and Patient Experiences with Voice-Controlled Intelligent Personal Assistants. Nurse Leader. 2023;21(2):252–258. doi: 10.1016/j.mnl.2022.06.009

[61] Mahdi SS, Battineni G, Khawaja M, Allana R, Siddiqui MK, Agha D. How does artificial intelligence impact digital healthcare initiatives? A review of AI applications in dental healthcare. Int J Inf Manag Data Insights. 2023;3(1):100144. doi: 10.1016/j.jjimei.2022.100144

