# Supplementary material for: Acceptance of voice assistant technology in dental practice: A cross sectional study with dentists and validation using structural equation modeling
Source: PLOS Digit Health. 2024 May 14;3(5):e0000510. doi: 10.1371/journal.pdig.0000510 (PMC11093337; doi:10.1371/journal.pdig.0000510)
Supplement: S1 Appendix — (DOCX) [file pdig.0000510.s001.docx]

S1 Appendix: Invitation Email to Participate in Study

Dear Dentist,

We are writing to invite you to participate in a research project that has the objective of evaluating the perceptions and beliefs of Ohio dentists about voice assistant technology.  Examples of voice assistant technology include Apple’s Siri, Amazon’s Alexa, and Google Assistant. Your responses to the survey will help guide the development of a voice assistant technology, which will allow dental charting using voice commands in a manner similar to Siri, Alexa, and Google Assistant.

All information provided will be anonymous and will only be used for conclusions in this survey. Your responses to the survey will not be tied in any way to your email or name. There are no foreseeable risks to participating in this research study.  There will be no personal benefit to participating in this study, other than contributing to development of this novel software.  This survey is voluntary and you may choose not to participate. If you agree to participate in this study, you may stop participating at any time.

Your participation in this project is greatly appreciated.  If you have any questions about the research study itself, please contact:

Dr. Spencer Warren at spencer.warren@nationwidechildrens.org

To access the survey instrument, please click on the following link: <link removed>

Thank you for your participation!
