# Supplementary material for: Acceptance of voice assistant technology in dental practice: A cross sectional study with dentists and validation using structural equation modeling
Source: PLOS Digit Health. 2024 May 14;3(5):e0000510. doi: 10.1371/journal.pdig.0000510 (PMC11093337; doi:10.1371/journal.pdig.0000510)
Supplement: S2 Appendix — (DOCX) [file pdig.0000510.s002.docx]

S2 Appendix: Consent Form

By completing this survey, you are agreeing to participate in this study. Disclaimer: Your participation in this survey is voluntary. All responses will be kept confidential. The survey should take 10 minutes to complete. Description of Voice assistant technology for this Study: Voice assistant technology utilizes voice commands and communication to accomplish different tasks on computers and electronic smart devices. Common examples of voice assistant technology include Amazon Alexa, Google Assistant, and Siri. Voice assistant technology is becoming more advanced and ubiquitous, and it may become a common tool in dental offices in the future. Patient information is commonly placed into the dental chart through mouse and keyboard by dental assistants and hygienists, but voice assistant technology would allow the dentist to directly enter patient information into the chart using voice commands. Voice assistant technology has the potential to increase the efficiency and accuracy of entering patient information during dental charting, such as charting caries location on a tooth chart. Voice assistant technology also has the potential to allow handsfree navigation of dental charts, such as navigating between x-rays and the tooth chart during a procedure, thus improving sanitation and decreasing workload for dental assistants and hygienists. This study’s aim is to assess the perceived usefulness and comfortability of utilizing voice assistant technology. CONSENT TO PARTICIPATE IN A CLINICAL RESEARCH STUDY STUDY TITLE: Evaluation of dentists’ perceptions and acceptance of using voice assistant software. PRINCIPAL INVESTIGATORS: Daniel Claman, DDS CONTACT TELEPHONE NUMBER: 931-389-1769 (24 hours a day, 7 days a week) NOTE: The words “you” and “your” are used in this consent form. These words refer to the study volunteer whether a child or an adult. 1) INTRODUCTION We invite you to be in this research study. Using this form as a guide, we will explain the study to you. If you have any questions about the study, please ask. If you do not want to be in this study, all regular and standard medical care will still be available to you here at Nationwide Children’s Hospital. Participation is voluntary. You can leave this study at any time. You will be given a copy of this form.2) WHERE WILL THE STUDY BE DONE AND HOW MANY SUBJECTS WILL TAKE PART? This study will be done at Nationwide Children’s Hospital, and we hope to enroll 7600 participants aged 22-90.3) WHAT WILL HAPPEN DURING THE STUDY AND HOW LONG WILL IT LAST? The purpose of this study is to evaluate dentists’ perceptions and intention to use voice assistant technology. This information will help in development of voice assistant software for dental electronic health records. Study participants: Subjects will complete a survey, once at the time of enrollment. 4) WHAT ARE THE RISKS OF BEING IN THIS STUDY? The risks for this study are no more than what happens in everyday life. Although we will take every precaution, there is a small chance of loss of confidentiality of your study information. There may be other risks of being in this research study that are not known at this time.5) ARE THERE BENEFITS TO TAKING PART IN THIS STUDY? Although there will be no benefit to you from being in this study, we hope to learn something that could help develop voice assistant software for dental practices in the future.6) WHAT ARE THE COSTS AND REIMBURSEMENTS? You will not be paid to participate in this study. 7) WHAT HAPPENS IF I DO NOT FINISH THIS STUDY? It is your choice to be in this study. You may decide to stop being in this study at any time. If you stop being in the study, there will be no penalty or loss of benefits to which you are otherwise entitled. If at any time the Principal Investigator believes that this study is not good for you, the study team will contact you about stopping. If the study instructions are not followed, participation in the study may also be stopped.8) OTHER IMPORTANT INFORMATION If you are an employee of Nationwide Children’s Hospital or the Research Institute at Nationwide Children’s Hospital, your job or performance appraisal will not be affected in any way if you decline to participate or withdraw your consent to participate in this study. Nationwide Children’s Hospital is a teaching hospital and we are committed to doing research. Doing research will enable us to learn and provide the best care for our patients and families. You may be asked to participate in other research studies in the future. You have the right to decide to participate or decline to participate in any future studies. We will not share your contact information with researchers outside Nationwide Children’s Hospital.9) HOW WILL MY STUDY INFORMATION BE KEPT PRIVATE? Efforts will be made to keep your study-related information confidential. However, there may be circumstances when this information must be released. For example, personal information regarding your participation in this study may be disclosed if required by state law. The results from this study may be published but your identity will not be revealed. 10) WHOM SHOULD I CALL IF I HAVE QUESTIONS OR PROBLEMS? If you have questions about anything while on this study or you have been injured by the research, you may contact the Principal Investigator at 931-389-1769, Monday – Friday. If you have questions, concerns, or complaints about the research; if you have questions about your rights as a research volunteer; if you cannot reach the Principal Investigator; or if you want to call someone else, call (614) 722-2708, Nationwide Children's Hospital Institutional Review Board, (the committee that reviews all research involving human subjects at Nationwide Children’s Hospital).
