# Supplementary material for: Acceptance of voice assistant technology in dental practice: A cross sectional study with dentists and validation using structural equation modeling
Source: PLOS Digit Health. 2024 May 14;3(5):e0000510. doi: 10.1371/journal.pdig.0000510 (PMC11093337; doi:10.1371/journal.pdig.0000510)
Supplement: S3 Appendix — (DOCX) [file pdig.0000510.s003.docx]

S3 Appendix: Demographic Questionnaire

1. When did you complete your dental education: < 5 years ago
2. 5-10 years ago
3. 11-15 years ago
4. 16-20 years ago
5. >20 years ago
6. Which of the following best describes you?
7. General Dentist
8. Pediatric Dentist
9. Orthodontist
10. Endodontist
11. Periodontist
12. Oral and Maxillofacial Surgeon
13. Prosthodontist
14. Oral and Maxillofacial Pathologist
15. Dental Anesthesiologist
16. Oral and Maxillofacial Radiologist
17. Dental Public Health
18. Other
19. If other, please explain
20. What is the location of your practice?
21. Rural
22. Suburban
23. Urban
24. Estimate what percentage of your patient population is insured by Medicaid/Medicaid HMO?
25. 0%
26. 1-24%
27. 25-50%
28. more than 50%
29. What is your age?
30. What is your gender?
31. Female
32. Male
33. Other
34. I prefer not to say
35. How would you identify yourself?
36. American Indian or Alaska Native
37. Asian
38. Black or African American
39. Native Hawaiian or Other Pacific Islander
40. White
41. Multi-race
42. Other
43. If other, please explain
44. What is your familiarity with voice assistant technology?
45. Never used
46. Used a few times
47. Use often
48. Use every day
