# Supplementary material for: Acceptance of voice assistant technology in dental practice: A cross sectional study with dentists and validation using structural equation modeling
Source: PLOS Digit Health. 2024 May 14;3(5):e0000510. doi: 10.1371/journal.pdig.0000510 (PMC11093337; doi:10.1371/journal.pdig.0000510)
Supplement: S4 Appendix — (DOCX) [file pdig.0000510.s004.docx]

S4 Appendix: Survey

5-Point Likert Scale Used:

1. Strongly Disagree
2. Disagree
3. Neutral
4. Agree
5. Strongly Agree

Effort Expectancy:

1. Learning how to use voice assistant technology is easy for me.
2. My interactions with voice assistant technology would be clear and understandable.
3. I find voice assistant technology easy to use
4. It is easy for me to become skillful at using voice assistant technology.
5. I would find it easy to get the voice assistant technology to do what I want it to do.

Performance Expectancy:

1. Using voice assistant technology would improve my daily life.
2. Using voice assistant technology increases my chances of achieving daily tasks that are important to me.
3. Using voice assistant technology would allow me to accomplish daily tasks more quickly.
4. Using voice assistant technology would enhance my effectiveness in completing daily tasks.

Perceived Enjoyment:

1. I find using voice assistant technology enjoyable.
2. The process of using voice assistant technology is pleasant.
3. I have fun using voice assistant technology.

Satisfaction:

1. I am very content with voice assistant technology.
2. I am very pleased with voice assistant technology.
3. I am satisfied with voice assistant technology’s efficiency.
4. I feel delighted with voice assistant technology.
5. Overall, I am satisfied with voice assistant technology.

Trust:

1. I believe that voice assistant technology is trustworthy.
2. I trust in voice assistant technology.
3. I do not doubt the honesty of voice assistant technology.
4. Even if not monitored, I would trust voice assistant technology to do the job right.
5. Voice assistant technology has the ability to fulfill its task.

Self-Efficacy:

1. I am confident in using voice assistant technology, even if there is no one around to show me how to use it.
2. I am confident in using voice assistant technology, even if I have never used such a system.
3. I am confident in using voice assistant technology, even if I have only the software manuals for reference.

Perceived Risk:

1. I think using voice assistant technology puts my privacy at risk.
2. Using voice assistant technology exposes me to an overall privacy risk.
3. Using voice assistant technology will not fit well with my self-image.

Behavior Intention:

1. Assuming I had access to voice assistant technology, I intend to use it.
2. Given that I had access to voice assistant technology, I predict that I would use it.
3. I plan to use voice assistant technology in the future.
